# GLUT4 Traffic through an ESCRT-III-Dependent Sorting Compartment in Adipocytes

**DOI:** 10.1371/journal.pone.0044141

**Published:** 2012-09-25

**Authors:** Françoise Koumanov, Vinit J. Pereira, Paul R. Whitley, Geoffrey D. Holman

**Affiliations:** Department of Biology and Biochemistry, University of Bath, Bath, United Kingdom; University of Geneva, Switzerland

## Abstract

In insulin target tissues, GLUT4 is known to traffic through multiple compartments that may involve ubiquitin- and/or SUMO-dependent targeting. During these trafficking steps, GLUT4 is sorted into a storage reservoir compartment that is acutely released by insulin signalling processes that are downstream of PI 3-kinase associated changes in inositol phospholipids. As ESCRT components have recently been found to influence cellular sorting processes that are related to changes in both ubiquitination and inositol phospholipids, we have examined whether GLUT4 traffic is routed through ESCRT dependent sorting steps. Introduction of the dominant negative inhibitory constructs of the ESCRT-III components CHMP3 (CHMP3(1–179)) and Vps4 (GFP-Vps4^E235Q^) into rat adipocytes leads to the accumulation of GLUT4 in large, coalesced and extended vesicles structures that co-localise with the inhibitory constructs over large parts of the extended structure. A new swollen hybrid and extensively ubiquitinated compartment is produced in which GLUT4 co-localises more extensively with the endosomal markers including EEA1 and transferrin receptors but also with the TGN marker syntaxin6. These perturbations are associated with failure of insulin action on GLUT4 traffic to the cell surface and suggest impairment in an ESCRT-dependent sorting step used for GLUT4 traffic to its specialised reservoir compartment.

## Introduction

The Endosomal Sorting Complex Required for Transport (ESCRT) is essential for membrane compartment and membrane protein organisation [1]. ESCRT proteins are conserved in organisms ranging from archaea to eukaryotes where they fulfil a range of diverse roles [1], [2]. The ESCRT system has been implicated in processes that include lysosome biogenesis via multivesicular body (MVB) formation [3], cytokinesis [4], [5], enveloped virus budding [6] and autophagy [7]. A common functional role for the protein components of the system is the deformation of membrane lipids and the generation of invaginated membrane structures including membrane tubes, buds and multivesicular endosomes (MVE) [1]. The complexes are also involved in membrane protein sorting and with selection of cargo membrane proteins for degradation, recycling to the plasma membrane or the trans-Golgi network (TGN).

The four ESCRT complexes include ESCRT-0, ESCRT-I, ESCRT-II and ESCRT-III which are recruited sequentially to membranes, particularly membranes of the endosome system that are rich in phosphatidylinositol 3-phosphate (PI(3)P). Components of ESCRT-0 have ubiquitin interacting motifs (UIM) and ubiquitin binding domains (VHS) and these are thought to facilitate the gathering of ubiquitinated cargo proteins into membrane patches [8]. ESCRT-I and ESCRT-II continue the process of concentrating membrane proteins while Charged Multivesicular Body Protein (CHMP) components of ESCRT-III allow membrane sorting and membrane deformation. The CHMP proteins of ESCRT-III include CHMP4, CHMP3 and CHMP2. These proteins can be autoinhibited through interactions between their N- and C-terminal domains [9], [10]. Removal of this autoinhibition allows the separate roles of the N- and C-termini to be manifest [11]. C-terminal regions of both CHMP3 and CHMP4 bind the ESCRT-III regulator Vps4 [12] and the de-ubiquitinating hydrolases (DUBs) including AMSH [9], [13]. The CHMP protein positively charged N-terminal regions interact with negatively charged phosphoinositides including phosphatidylinositol 3,5-bisphosphate (PI(3,5)P_2_) and this association may allow a number of specific lipid targeting processes [11], [14].

ESCRT dependent selection of cargo appears to be associated with ubiquitination cycles involving ubiquitin ligases and DUBs [15], [16] (including AMSH and USP8 in mammals [13]). Similar considerations probably apply to the handling of SUMOylated cargos, including Top1, by ESCRT proteins [17], but this has not been widely studied. The sorting role for ubiquitin-like domains in GLUT4 traffic is beginning to emerge from recent studies [18]–[22] but the possibility of ESCRT-dependent sorting of the tagged GLUT4 has not been previously addressed. Here we provide evidence that GLUT4 is routed through an ESCRT compartment in insulin-target cells and that perturbation of this traffic leads to a failure of GLUT4 to reach its normal intracellular storage vesicle compartment (GSVs) and by an inability of GLUT4 to be recruited to the cell surface upon insulin stimulation.

## Materials and Methods

### DNA constructs

pCis2 HA-GLUT4 was a gift from Dr. Samuel Cushman and has been described previously [23]. pEGFP-C1-VPS4, pEGFP-C1-VPS4^E235Q^ and pEGFP-N1-CHMP3^1–179^ constructs have been described previously [5], [11].

### Antibodies

Rabbit polyclonal GLUT4 antibody was raised against a GLUT4 C-terminal peptide [24]. Mouse anti HA antibody (Clone 16B12) was purchased from Covance, mouse anti-EEA1 antibody from BD Biosciences, mouse anti-Ubiquitin antibody (Clone FK2) from BIOMOL, mouse anti-Syntaxin 6 antibody from BD Biosciences and mouse anti-Transferrin receptor (TfR) antibody from Zymed. AlexaFluor 546 conjugated goat anti-mouse IgG and Alexa Fluor 633 conjugated goat anti-rabbit IgG were from Molecular Probes. Mouse IgG secondary antibody β-galactosidase conjugate was from SouthernBiotech.

### Isolation of primary rat adipocytes

Adipose cells from epididymal fat pads of male Wistar rats, weighing 180–200 g, were prepared by collagenase digestion as described previously [25]. Cells were maintained at 37°C in Krebs-Ringer-HEPES (KRH) buffer (140 mM NaCl, 4.7 mM KCl, 2.5 mM CaCl_2_, 1.25 mM MgSO_4_, 2.5 mM NaH_2_PO_4_, 10 mM HEPES, (pH 7.4)) with 1% (w/v) bovine serum albumin (BSA) and 200 nM adenosine. Before transfection the cells were washed twice with DMEM supplemented with 200 nM adenosine and brought to a 50% cytocrit.

### Adipocyte transfection

Rat adipocytes were electroporated with pCis2 HA-GLUT4 alone or together with pEGFP-VPS4, pEGFP-VPS4^E235Q^ or pEGFP-CHMP3^1–179^ according to the method described by Al-Hasani et al. [23]. Briefly, 200 μl of 50 % cytocrit rat adipocytes were added to 200 μl of DMEM containing 100 μg of carrier DNA (hearing sperm DNA, Promega) and 0.1 μg of pCis2 HA-GLUT4 alone or together with 0.8 μg of pEGFP-VPS4, pEGFP-VPS4^E235Q^ or pEGFP-CHMP3^1–179^. The amounts of HA-GLUT4 and inhibitory construct cDNA were optimised as described [23]. Electroporation was carried out using the BioRad Gene pulser with a capacitance extender attached in 0.4-cm gap-width cuvettes (Bio-Rad). Each cuvette was electroporated once at 400 V, 500 μF. Cells from 4 to 5 cuvettes were pooled together, washed once with DMEM supplemented with 200 nM adenosine and then resuspended in DMEM supplemented with 3.5 % BSA and 200 nM adenosine. The transfected adipocytes were incubated for 5 h at 37°C. After washing in KRH buffer supplemented with 1% BSA and 200 nM adenosine cells were left un-stimulated or stimulated with 60 nM insulin for 20 min at 37°C. Using the above described electroporation conditions we obtained 20 to 25 % transfection efficiency as estimated by examining the cells by fluorescent microscopy and recording GFP positive cells.

### Determination of HA-GLUT4 antibody binding

Transfected rat adipocytes, left un-stimulated or stimulated with 60 nM insulin, were incubated in presence of 2 mM KCN for 3 min to stop GLUT4 recycling. Cells were then incubated with 1 μg/ml anti-HA antibody in Krebs-Ringer-HEPES buffer supplemented with 5% BSA and 200 nM adenosine for 1 h at room temperature with occasional mixing. After three washes in Krebs-Ringer-HEPES buffer supplemented with 5% BSA and 200 nM adenosine, adipocytes were incubated with 1 μg/ml anti-mouse IgG secondary antibody β-galactosidase conjugate. Cells were then washed in Krebs-Ringer-HEPES buffer supplemented with 200 nM adenosine 4 times and 10 μl of cell suspension were added in quadruplicated in black 96 well plates (Fluotrac 200, Greiner). Fluorescein digalactosidase (FDG) at a final concentration of 0.1 mM in Krebs-Ringer-HEPES buffer was added to each well. The rates of fluorescence generated per mg protein were then determined from measurements (for an hour at 15 sec intervals) in a Pherastar (FS) fluorescent plate reader (BMG) at 520 nm. An aliquot of cells from each condition was analyzed by immuno-blotting to assess and normalize for the level of expression of HA-GLUT4 cDNA.

### Indirect immunofluorescence microscopy

Stimulated adipocytes were fixed by incubation with 4% (w/v) paraformaldehyde in KRH buffer for 20 min at room temperature, and washed 3 times with PBS. Cells were then treated with permeabilisation buffer (0.1% saponin, 1% (w/v) BSA, 3% (v/v) goat serum in PBS) for 45 min. The co-localisation of the EGFP tagged constructs with GLUT4 and cell organelle markers was determined in adipocytes that were incubated with primary antibodies diluted in permeabilisation buffer overnight at 22°C. The primary antibodies were used at the following dilutions: rabbit anti GLUT4 antibody (1∶1000), mouse anti HA antibody (1∶500), mouse anti-EEA1 antibody (1∶250), mouse anti-Ubiquitin antibody (1∶400), mouse anti-Syntaxin 6 antibody (1∶200) and mouse anti-Transferrin receptor antibody (1∶100). 18 hours later, the cells were washed in permeabilisation buffer, incubated with species-specific fluorophore conjugated secondary antibodies (1∶300 dilution) for 2 h at room temperature with a final wash step in permeabilisation buffer. Cells were mounted onto a glass coverslip with Vectashield mounting medium (Vector Laboratories).

Confocal microscopy was performed on a Zeiss LSM 510 META microscope with 63×1.4 NA oil-immersion objective and with dual or triple laser excitation at 458–488, 543 and 633 nm. Images (1024×942) of individual cells were saved as TIFF files using the Zeiss LSM Image analysis software and intensity levels of the individual channels were adjusted to comparable dynamic range [26] in Adobe Photoshop.

To measure the extent of enlargement of vesicles in which the fluorescent signals from EGFP-VPS4^E235Q^, endogenous GLUT4 and endosomal markers co-localised, individual cells were analysed with the Measure tool in the ImageJ National Institute of Health software (http://imagej.nih.gov/ij/). Individual enlarged vesicles with a visible lumen were selected using the circular tool to define the regions of interest and the areas (in pixels) of these structures were then determined. As a control, the areas of vesicles positive for GLUT4 or for ubiquitin or for the endosomal markers in cells transfected with pEGFP-Vps4 were measured. On average 5 to 10 enlarged structures and 15 to 20 control structures were measured per cell. Data were presented as average values from 10 to 14 individual cells. Statistical significance was calculated using two-tailed unpaired t-test. P values of less than 0.05 were considered significant.

## Results

### ESCRT-III constructs inhibit insulin-stimulated HA-GLUT4 translocation in rat adipocytes

We have used two ESCRT constructs to investigate the dependence of GLUT4 translocation on movement through the ESCRT pathway in insulin-stimulated adipocytes. The first construct encodes the N-terminal portion of CHMP3, amino acids 1 to 179. We have previously developed this N-terminal construct as a dominant negative inhibitor of ESCRT-III function [5]. The construct has an open conformation and is not autoinhibited by the C-terminal domain. This construct has been shown to bind to membranes and to induce an enlarged endosomal compartment. The second construct, GFP-Vps4^E235Q^, encodes the dominant-negative mutant of the AAA ATPase Vps4 which is deficient in ATPase activity, and consequently stops the disassembly of the ESCRT-III components. The usual cellular change induced by this construct includes the generation of enlarged endosomal vesicles.

**Figure 1:**
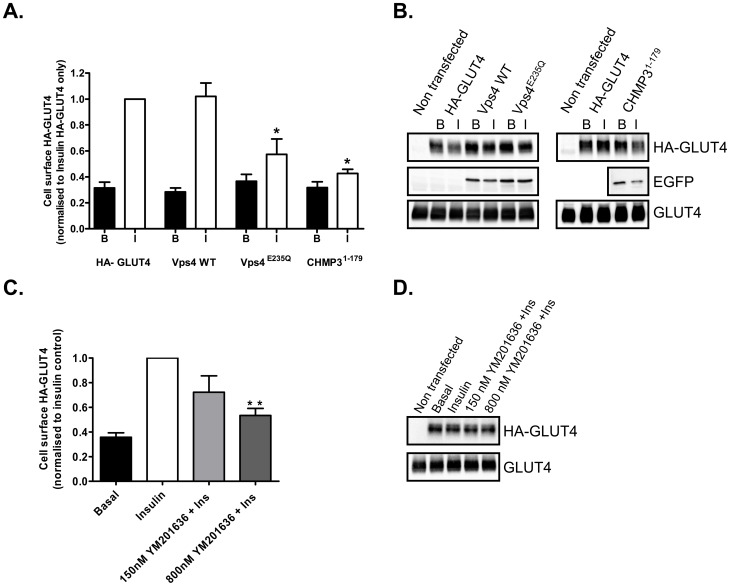
ESCRT-III constructs lead to inhibition of GLUT4 translocation to the cell surface of rat adipose cells. (A) Primary rat adipocytes were transfected by electroporation with pCis HA-GLUT4 and pEGFP VPS4, pEGFP VPS4^E235Q^ or pEGFP CHMP3^1–179^ and then maintained in culture for 5 h. HA-GLUT4 at the cell surface was detected with anti-HA antibody and β-galactosidase conjugated secondary antibody. The signal was measured with a fluorescent β-galactosidase substrate. Results are mean and SEM from 3 independent experiments. * p<0.05 (comparison of pCis HA-GLUT4 only control *vs*. pCis HA-GLUT4 co-transfected with ESCRT-III pEGFP constructs). (B) Representative immunoblots for the levels of expression of HA-GLUT4, EGFP-Vps4, EGFP-Vps4^E235Q^, CHMP3^1–179^-EGFP and total GLUT4 in the transfected rat adipocytes. (C) The PIKfyve inhibitor YM201636 decreases GLUT4 translocation to the cell surface in a dose dependent manner. Primary rat adipocytes were transfected by electroporation with pCis HA-GLUT4 and then maintained in culture for 5 h. Cells were left untreated or incubated with 150 nM or 800 nM YM201636 for 30 min prior to insulin stimulation. HA-GLUT4 at the cell surface was detected with anti-HA antibody and β-galactosidase conjugated secondary antibody. The signal was measured with a fluorescent β-galactosidase substrate. Results are mean and SEM from 3 independent experiments. ** p<0.01 (comparison of Insulin stimulated control cells *vs*. YM201636 treated cells prior to insulin stimulation). (D) Representative immunoblots for the levels of expression of HA-GLUT4 and total GLUT4 in the transfected rat adipocytes treated with YM201636.

EGFP versions of these constructs were co-transfected into primary rat adipocytes together with HA-tagged GLUT4 [23] used as a reporter. The effects of GFP-Vps4^E235Q^ and CHMP3^1–179^GFP on insulin-stimulated GLUT4 translocation were followed by measuring the amount of HA-tag present at the surface of intact cells. Both constructs decreased insulin-stimulated GLUT4 translocation by 70–80% without significantly affecting the basal levels of GLUT4 present at the cell surface (Figure 1A). Expression of the wild-type GFP-Vps4 construct had no effect on insulin-stimulated HA-GLUT4 translocation to the cell surface. Wild-type CHMP3-GFP could not be used as a control in this experiments as over-expression of the wild-type protein has been reported to induce an enlarged phenotype [27]. Immunoblotting analysis indicated that the HA-GLUT4 was expressed to a similar level in all conditions and that co-expression of the GFP-tagged ESCRT constructs did not affect the level of expression of the HA tagged GLUT4 (Figure 1B).

**Figure 2:**
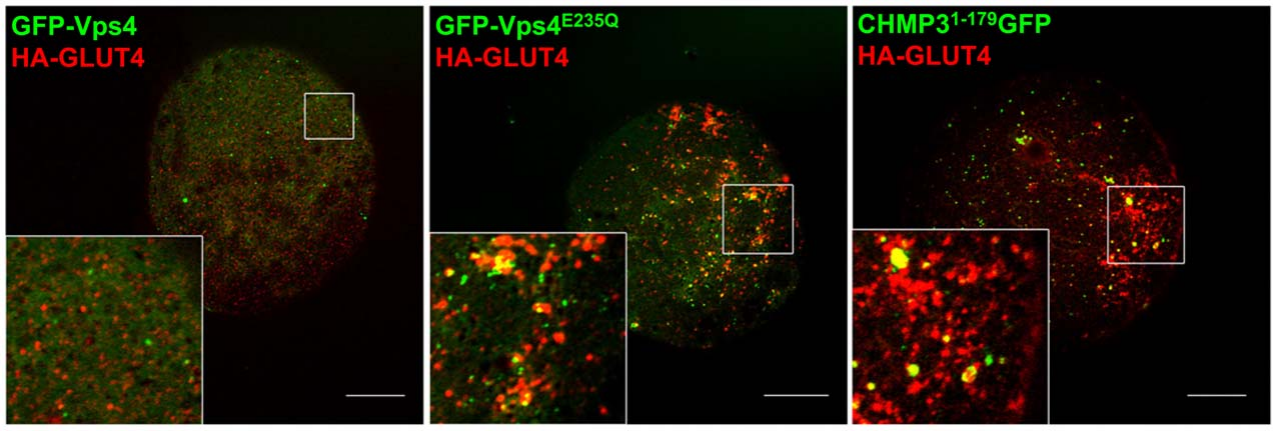
ESCRT-III constructs lead to swollen and extended GLUT4 compartments in rat adipose cells. Confocal microscopy examination of primary rat adipocytes co-transfected with HA-GLUT4 (red) and EGFP-tagged Vps4 WT, Vps4^E235Q^ or CHMP3^1–179^ (green) and stimulated with 60 nM insulin for 20 min**.** Cells were fixed with 4% paraformaldehyde, permeabilised in 0.1% saponin and immuno-stained with anti-HA antibody and anti-mouse IgG-Alexa 633 secondary antibody. Images were acquired with LSM510 Meta confocal laser scanning microscope and are from single adipose cells representative of the cell populations from at least three separate experiments. Bars 20 μm.

Localisation studies (Figure 2) revealed that the dominant negative, but not the wild-type, GFP-Vps constructs generate a distinct GFP-positive punctate staining of enlarged and swollen structures throughout the cell. In the absence of the dominant negative constructs GLUT4 is distributed widely and most of the GLUT4 is present in small punctuate spots. This is typical of mature rat fat cell GLUT4 [28], but typically GLUT4 distribution in the adipose cell line 3T3-L1 is a mixture of dispersed vesicular structures and a larger perinuclear compartment. Following the perturbation of ESCRT-III, much of the HA-GLUT4 became localised to enlarged coalesced-vesicular structures which partially co-localised with the ESCRT proteins. The extent of co-localisation of GLUT4 and the GFP-Vps4^E235Q^ in the enlarged vesicular structures was variable and in many cases these two proteins appeared to be localised to the same swollen structures but to non-overlapping and distinct sub-regions of these structures (Figure 2).

As CHMP3 (and possibly other ESCRT components) interact with PI(3,5)P_2_ [11] we have examined whether inhibition of PI(3,5)P_2_ production with the PIKfyve inhibitor YM201636 also reduces the insulin-stimulated translocation of HA-GLUT4 to the cell surface of adipocytes. YM201636 at concentrations previously reported to inhibit PIKyve [29] significantly inhibit translocation of GLUT4 (Figure 1 C, D). However, we did not observe (data not shown) the formation of an enlarged compartment comparable with that observed following introduction of Vps4^E235Q^ and CHMP3^1–179^.

### ESCRT-III perturbation induces the formation of a hybrid compartment

**Figure 3:**
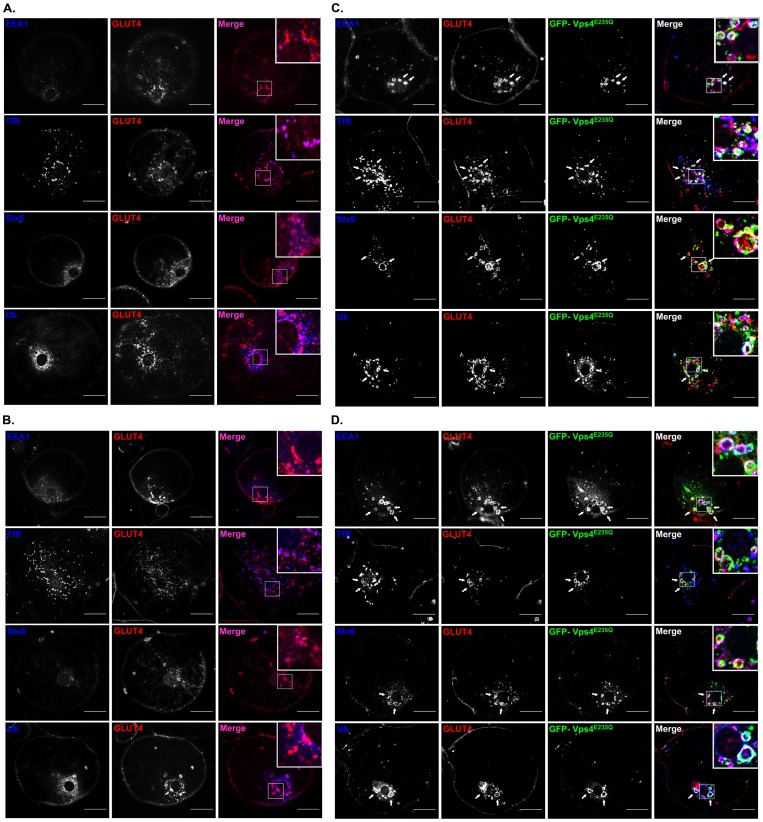
ESCRT-III constructs lead to formation of a hybrid compartment in which endosomal markers co-localise with GLUT4. The distribution of endogenous GLUT4 was detected with a rabbit polyclonal anti GLUT4 C-terminal peptide antibody. GLUT4 localization (red in merged images) was compared with EEA1, ubiquitin, syntaxin6 and transferrin receptors (blue in merged images) using the antibodies described in the Materials and Methods section. Endosomal marker distributions in the presence of non-perturbing wild-type GFP-Vps4 in the basal state (**A**) and the insulin stimulated state (**B**). The EGFP-Vps4 is highly dispersed and is cytosolic under these conditions (not shown for clarity). Expression of the EGFP-Vps4^E235Q^ (green in merged images) leads GLUT4 vesicle coalescence to form swollen compartments. The endosomal markers EEA1, transferrin receptors (TfR), syntaxin6 (Stx6) and ubiquitin (Ub) colocalise with both endogenous GLUT4 and with EGFP-Vps4^E235Q^ both in the basal state (**C**) and the insulin stimulated state (**D**). Images were acquired with LSM510 Meta confocal laser scanning microscope and are from single adipose cells representative of the cell populations from at least three separate experiments. Bars 20 μm. Arrows point at hybrid enlarged tubulo- vesicular structures.

In the absence of ESCRT-III perturbation and in basal cells, endosome marker experiments indicate that GLUT4 is sequestered in a cellular compartment in which there is extensive co-localisation with Syntaxin6. Syntaxin6 localisation defines a TGN-like compartment but is distinct from compartments in which TfR and EEA1 are highly localised (Figure 3A). Following insulin stimulation of these cells GLUT4 becomes more localised at the cell surface and just below it (Figure 3B). These observations are consistent with previous studies on rat fat cells [28], [30]–[32].

Following the expression of the GFP-Vps4^E235Q^ construct GLUT4 becomes more extensively localised with the syntaxin6, EEA1 and transferrin receptor in the swollen ESCRT-III compartment (Figure 3 C,D). This suggests the formation of a hybrid compartments in which there is merging, or lack of sorting, of distinct endosome and TGN protein components. Under these conditions, there are no significant differences between basal and insulin-stimulated cells in the distributions of syntaxin6, EEA1 or transferrin receptors.

In the absence of ESCRT-III perturbation the levels of co-localisation of GLUT4 and ubiquitin are low (Figure 3 A,B). This is partly a consequence of low and indistinct signal from the cytosol and small membrane particles. The extent to which ubiquitinated proteins cluster on enlarged membrane structures is increased in the presence of the GFP-Vps4^E235Q^ and there is also increased co-localisation with GLUT4 and the co-associated markers that reveal a hybrid endosome-TGN compartment (Figure 3 C,D). Analysis of individual cells positive for the GFP-Vps4^E235Q^ signal revealed that on average 7.69±0.60 enlarged vesicles with a visible lumen and positive for GFP-Vps4^E235Q^, GLUT4, ubiquitin or the endosomal/TGN markers were detected in the perinuclear region of non-stimulated cells. Similarly, in insulin-stimulated cells positive for GFP-Vps^E235Q^ 7.37±0.56 enlarged vesicles were observed per cell. The relative areas of these enlarged structures were determined with the Measure tool in the ImageJ software. This analysis revealed a 10-fold increase in the pixel areas of the enlarged structures compared to the control structures (Table 1). No differences were observed in the sizes of the enlarged structures in comparing un-stimulated with insulin-stimulated cells. Using the area measurement tool in the LSM imaging software the areas of the enlarged compartments in basal or insulin-stimulated cells were estimated to be 14.00±1.74 μm^2^ and 18.04±1.79 μm^2^ respectively (Table 1). The areas of control vesicles could not be accurately calculated because of the limits of light microscopy resolution. The average diameter of the hybrid structures was 2.40±0.14 μm for unstimulated and 2.71±0.18 μm for insulin-stimulated cells.

**Table 1:** Enlargement of a coalesced tubulo-vesicular structure in adipocytes treated with ESCRT-III constructs.

	EGFP-Vps4		EGFP-Vps4^E235Q^	
	Basal	Insulin	Basal	Insulin
**Area (pixels)**	85.92±24.11	131.57±5.37	1411.61±183.32^***^	1448.52±168.90^***^
**Area (μm^2^)**	-	-	14.00±1.74	18.04±1.80
**Diameter (μm)**	-	-	2.40±0.14	2.71±0.18

The areas of enlarged vesicular structures, with visible lumens and positive for EGFP, GLUT4 and ubiquitin or the endosomal markers, were measured in pEGFP VPS4^E235Q^ and in wild-type pEGFP-Vps4 transfected adipocytes (as described in the Materials and Methods section). Results are mean and SEM from 10 to 14 individual cells. *** p<0.001 (enlarged *vs* control vesicles). Only the large pixel areas from the enlarged structures could be accurately converted to μm^2^_._

**Figure 4:**
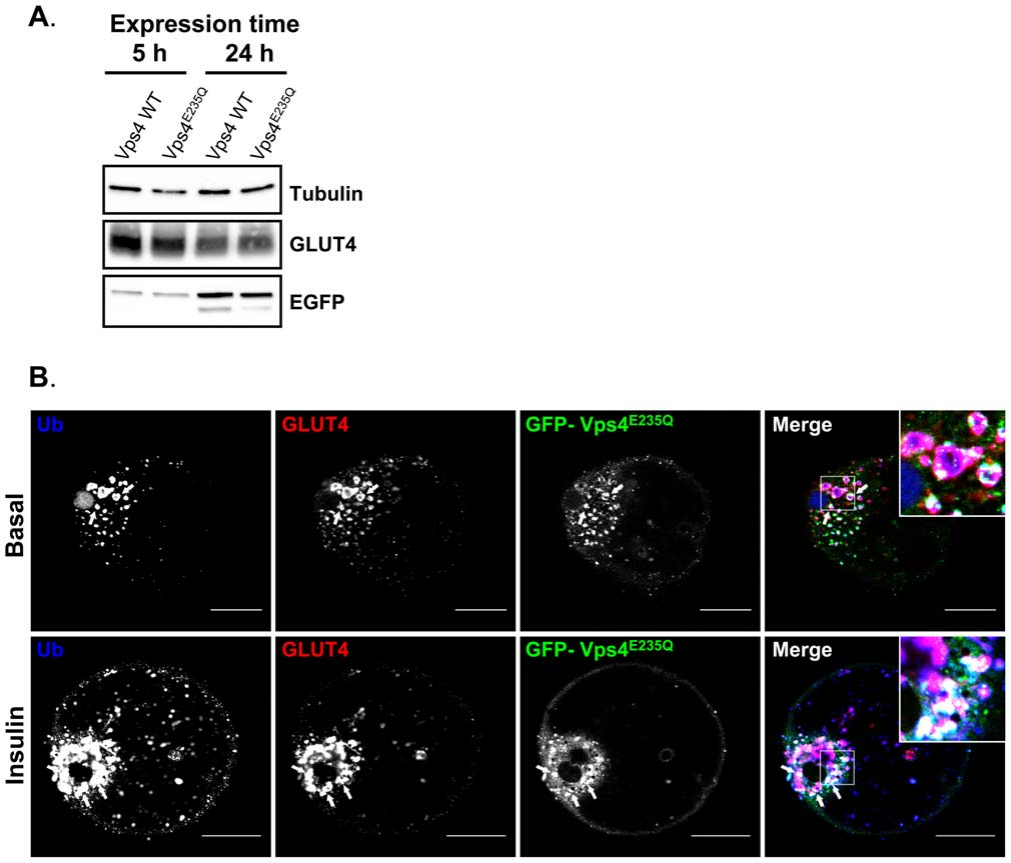
24-hour expression of ESCRT-III constructs increases the intracellular accumulation of GLUT4 in a coalesced enlarged compartment positive for ubiquitin. (A) Immunoblot analysis of the levels of expression of EGFP-Vps4 and EGFP-Vps4^E235Q^ in rat adipocytes transfected and maintained in culture for 5 h or 24 h. (B) Confocal microscopy examination of primary rat adipocytes transfected with EGFP-tagged Vps4 WT or Vps4^E235Q^ and maintained in culture for 24 h. Ubiquitin (Ub) colocalises with endogenous GLUT4 and with EGFP-Vps4^E235Q^ after 24 h expression in both basal and insulin-stimulated cells. Images were acquired with LSM510 Meta confocal laser scanning microscope and are from single adipose cells representative of the cell populations from at tubulo- vesicular least two separate experiments. Bars 20 μm. Arrows point at hybrid enlarged structures.

In order to determine if extended expression of the ESCRT-III construct would further affect GLUT4 distribution we compared rat adipocytes expressing GFP-Vps4 or GFP-Vps4^E235Q^ for 5 h or 24 h. Immunoblot analysis revealed an increase expression of GFP-Vps4 and GFP-Vps4^E235Q^ after 24 h expression (Figure 4A). Immunofluorescent analysis revealed that after 24 h expression, GLUT4 and ubiquitin colocalise extensively with the increased GFP-Vps4^E235Q^ (Figure 4B). The distribution of GLUT4 is very similar to that observed after 5 h expression. We conclude that GLUT4 is completely trapped in the enlarged endosomal compartment by the 5 h treatment. As the onset of the trapping is relatively rapid we cannot make an inference as to whether GLUT4 is first moved to the PM before transit to a sorting hub for generation of GSV.

## Discussion

GLUT4 proteins are sorted to insulin-responsive GLUT4 storage vesicles from which they are released to the cell surface upon insulin stimulation. The signals involved in sorting GLUT4 to GSV's are complex and various intracellular trafficking itineraries have been proposed [33]. However, the involvement of an ESCRT-dependent step in this traffic has not been previously considered. We report here that perturbations of the ESCRT machinery result in reduced levels of GLUT4 translocation to the plasma membrane in response to insulin. Furthermore, we show that disrupting the function of the ESCRT machinery leads to extensive accumulation of GLUT4 on intracellular membranes containing endosomal markers and the TGN marker syntaxin6 suggesting an accumulation in a hybrid enlarged tubulo- and multivesicular compartment. This suggests that a large proportion of GLUT4 is trapped by ESCRT perturbation and is unable to gain access to an insulin-responsive compartment. In comparison with unperturbed GLUT4 intracellular vesicle compartments, the enlarged GLUT4 compartment produced by ESCRT perturbation is highly enriched in ubiquitinated proteins. This suggests that, like other cargos that are routed through multivesicular endosomes, GLUT4 may undergo changes in tagging with ubiquitin-like domains or associate with chaperone proteins that are ubiquitin tagged [8].

Insulin action in adipocytes is highly dependent on inositol lipid signalling and perturbations in the levels of the phosphatidyl-inositides, mainly phosphatidylinositol 3,4,5-trisphosphate (PIP3), have been shown to be associated with formation of extended, enlarged and vacuolated GLUT4 compartments [34]. Studies on insulin-regulated GLUT4 traffic have led to the well-supported proposal that signalling is mediated through activation of Class1A PI 3-kinases and increases in PIP3. However, levels of PI(3,5)P_2_ are also increased by insulin signalling via activation of Class2 and Class3 PI 3-kinases to produce PI(3)P and by activation of PIKfyve [35]–[37]. It is therefore of note that ESCRT traffic is highly dependent on CHMP3 which has been shown to bind inositol lipids, particularly PI(3,5)P_2_. We observed inhibition of GLUT4 traffic following treatment with a PIKfyve inhibitor but we did not observe a marked enlargement of endosomes. We cannot therefore directly correlate the binding of CHMP3 to PI(3,5)P_2_ or to the function of PIKfyve. An enlarged endosome phenotype does occur in several other cell types following treatment with the PIKfyve inhibitor [29], [38]. By contrast, the morphological changes associated with inhibition of PIKfyve are generally not evident in adipocytes [39]. Furthermore, the kinase dead PIKfyve mutant does not induce an enlarged endosome phenotype in 3T3-L1 adipocytes [40]. This may mean that in adipocytes reduction in PI(3,5)P_2_ alone is insufficient to fully perturb ESCRT function. It will be of interest in future to determine the extent to which the traffic of GLUT4 through the ESCRT compartment is dependent on, or influenced by, insulin dependent changes in both PIP3 and PI(3,5)P_2_. Similarly, silencing of CHMP3 with siRNA treatment HeLa cells does not produce enlargement of endosomes and the endosome enlargement effect is confined to the dominant negative CHMP3 [41], [42].

It has recently been reported that GLUT4 is ubiquitinated and that this modification is necessary for its sorting into an insulin responsive compartment [22]. However, only a very low percentage (0.1%) of GLUT4 is reported to be ubiquitinated at steady-state. In our studies, we were unable to detect this low level of GLUT4 ubiquitination using immunoprecipitation of HA-tagged GLUT4 followed by ubiquitin detection by western blot. However, GLUT4 may associate with other proteins that may be tagged with ubiquitin-like domains. The genesis of this compartment is dependent on GGA adapters that bind to sortilin on the GSVs. Additional proteins associated with this compartment include TUG (Tether containing UBX domain for GLUT4). UBX (ubiquitin associated) domains of TUG (which are similar in structure to ubiquitin) are thought to retain the GSV and a construct of the UBX domain can lead to release of the GSVs from an intracellular storage location to the cell surface [43], [44]. However, the mechanism by which insulin action leads to a change in the GSV-TUG interaction is currently unknown. Direct SUMOylation of GLUT4 has been reported to occur at quite high levels [18], [19], [45]. In addition GLUT4 can associate with the Ubc9, a SUMO conjugating enzyme [18] and with another SUMOylated protein Daxx [19]. All these studies point towards a complex array of processing of GLUT4 in conjunction with ubiquitin-containing or ubiquitin-like protein domains and it will be of interest in future to determine how much of this processing is dependent on the ESCRT complexes.

**Figure 5:**
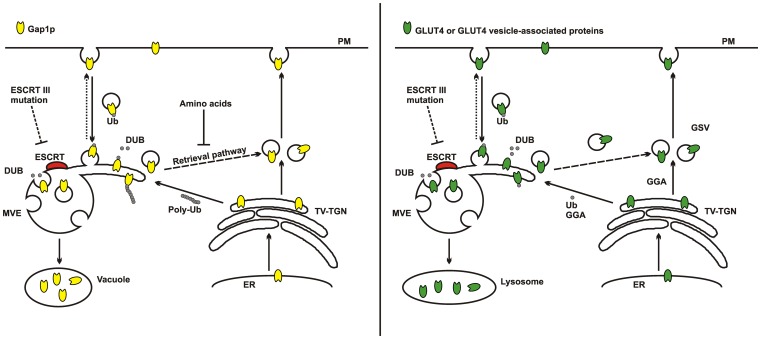
A model depicting the parallel between Gap1p trafficking in yeast and insulin-sensitive GLUT4 trafficking in mammalian cells. Left panel: Amino acid regulated Gap1p trafficking in yeast. In response to fluctuations in amino acid levels Gap1p traffics to the vacuole for degradation or to the plasma membrane (PM). This trafficking is regulated by Gap1p ubiquitination (Ub) and is dependent on ubiquitin ligases and deubiquitinating hydrolases (DUB). Before it reaches the vacuole Gap1p is sorted to the multivesicular endosomes (MVE), where, depending on the amino acid environment, it will either be degraded or redirected to the PM directly or via the tubulo-vesicular TGN (TV-TGN). The ESCRT-III mutants have been reported to block Gap1p trafficking to the vacuole and instead Gap1p is redirected to the plasma membrane. Model adapted from that described by the Kaiser's group [47], [48] **Right panel: Insulin stimulated GLUT4 trafficking.** In response to insulin stimulation GLUT4 traffics from a storage compartment (GSV) to the plasma membrane (PM). The traffic of GLUT4 back to the GSVs is complex and requires ubiquitin (Ub) and is dependent on GGA proteins. It is currently unclear whether ubiquitinylation of GLUT4 or a GLUT4 vesicle resident protein is responsible for the extensive localization with Ub and GLUT4. In our study we report that ESCRT-III mutants block GLUT4 trafficking and trap it an enlarged hybrid compartment together with endosomal and TGN markers. We propose that ESCRT compartment is an extended tubulo-vesicular structure that may act as a hub that sorts GLUT4 that is destined either for degradation or for insulin-regulated traffic. ESCRT dependent membrane curvature machinery and associated DUB activity may facilitate the targeting of GLUT4 (see Discussion text for details). Such a model is consistent with a previously proposed model of endosomal maturation and formation of tubulo-vesicular MVE structures [3].

Parallels have been drawn between the traffic of GLUT4 and the yeast amino acid transporter Gap1p [22], [46]. This protein traffics between three main compartments, the TGN, the plasma membrane and the vacuole. The Gap1p storage compartment is associated with the TGN and residence in this compartment is dependent on GGA and on ubiquitination. Under nitrogen deficient conditions Gap1p is deubiquitinated and moves towards the plasma membrane. Under nitrogen rich conditions the ubiquitinated Gap1p is directed towards multivesicular endosomes (MVEs) and to the vacuole for degradation as it is no longer needed. In the case of GLUT4 it is proposed here that sequential ubiquitination and deubiquitination of GLUT4 (or a GLUT4 vesicle associated protein) are required for directing GLUT4 to the GSV compartment [22] rather than the plasma membrane (as in the Gap1 case). This distinction probably occurs because insulin action is required for movement from the GSV compartment to the plasma membrane (Figure 5). The comparisons with the Gap1p system [22], [47], [48] are relevant here as it has been determined that amongst the yeast mutants that have defective Gap1p traffic are ESCRT components including Vps24 (CHMP3) and Vps4. In this case ESCRT mutants lead to reduced vacuole targeting of Gap1p and increased traffic to the plasma membrane [47], [48]. Distinct roles of mono- and poly-ubiquitination have been demonstrated in studies on ESCRT dependent traffic between MVEs and the plasma membrane and between the TGN and the MVEs, respectively [47].

GLUT4 that accumulates in the MVE upon ESCRT perturbation may have previously entered from the plasma membrane or trafficked to the MVE from the TGN. The latter route is more consistent with the ubiquitin and GGA dependent sorting of GLUT4 [22], but this route may require polyubiquitination [47]. GLUT4 is relatively stable protein with a half life of 48 hours in 3T3L1 adipocytes [49], [50] and each GLUT4 molecule possibly gains access to the plasma membrane and endosomes multiple times before being eventually degraded, even in unstimulated cells [51]. GLUT4 is now known to follow an unusual retrograde trafficking route from endosomes to the TGN that involves the Clathrin Heavy Chain CHC22 rather than CHC17 [26], [52]. This route is distinct from the early endosome to TGN pathway. The CHC22 dependent step is downstream of the CHC17-dependent early endosome sorting step suggesting that some cargos, such as GLUT4, enter a “non-early” endosome compartment before retrograde traffic to the TGN. CHC22 deletion interferes with this step and causes GLUT4 loss, possibly due to GLUT4 degradation [26]. Furthermore, knockdown of CHC22 causes some increased tubulation of the GLUT4 [26] which is similar to the extended and coalesced GLUT4 compartment reported here and illustrated in a model form in Figure 5. GLUT4 may access this compartment so that it can either be conserved by retrograde traffic to the TGN-associated GSV compartment or alternatively degraded by following an MVE to lysosome route. Consistent with this possibility is the observation that residues close to the C-terminal LL targeting signals on GLUT4 and Gap1p are similar, but not identical, to those in the lysosome resident protein LIMPII [53]. Mutation of GLUT4 in the vicinity of the LL motif can lead to its traffic to the lysosome [53]. We envisage that the tubular-vesicular MVE structures are an immature MVB (as described [3]) which acts as a hub or sorting station. This hub structure would likely facilitate docking and fusion of incoming GLUT4 vesicles with outward-facing topology and allow budding of vesicles with cargos (such as GLUT4) not destined for degradation. Changes in lipid domains within the same MVE could then be a platform for ESCRT protein activity with production of an inward-facing topology of membrane domains that leads to the degradation route. Testing of the model described will require a dynamic and kinetic analysis of movement between the proposed MVE compartment and the GSV and plasma membrane compartments.

Membrane proteins such as EGFR and CXCR4 require deubiquitination by DUBs prior to sorting into MVEs [54], [55] and the accumulation of ubiquitinated proteins suggests a failure in the functional recruitment of DUBs to this compartment. Two DUBs, AMSH and USP8 have both been shown to associate with the ESCRT machinery via ESCRT-0 and ESCRT-III proteins [56]–[59]. In the case of the interactions with ESCRT-III, both DUBs contain MIT domains that interact with C-terminal MIT interacting motifs (MIMs) of a number of ESCRT-III proteins [60]. There is mounting evidence that spatial and temporal regulation of deubiquitination, rather than ubiquitination *per se*, may be crucial in determining the fate of ubiquitinated proteins at endosome, TGN and MVE sorting steps. For example AMSH and USP8 can have very different effect on the sorting of ubiquitinated membrane proteins. While AMSH is required for sorting of EGFR into MVEs and degradation in lysosomes [61], deubiquitination of EGFR by USP8 protects it from lysosomal degradation [54]. How the ESCRT machinery is involved in regulating ESCRT related DUB activity and alters the fate of cargo proteins needs to be explored in further detail. We hypothesise that by functionally disrupting the ESCRT machinery, and by interference with deubiquitination of GLUT4 (or GLUT4 associated proteins), a failure to sort GLUT4 to GSVs occurs (Figure 5 right panel). Both of the dominant negative constructs used in this study could interfere with the recruitment of MIT containing DUBs. The truncated CHMP3^1–179^GFP lacks its C-terminus which would normally contain its MIT interacting motif (MIM) [61]. GFP-Vps4^E235Q^ contains an MIT domain that binds to MIMs on ESCRT-III components [62]. This may prevent binding of MIT containing DUBs as this mutated form of Vps4 cannot be released from ESCRT-III due to defective ATPase activity. However, the DUB family is very large and unidentified DUBs may influence GLUT4 traffic during ESCRT-III sorting. It will be interesting to determine whether knock down of levels of specific DUBs will result in a failure of GLUT4 to sort to GSVs and thereby lead to decreased levels of GLUT4 at the plasma membrane in response to insulin. In addition, the possibility of ESCRT involvement in the perturbation of GLUT4 traffic that occurs in insulin-resistant cells remains to be explored.
